# A Pediatric Case Report of Acute Torticollis Secondary to Atraumatic Cerebellar Hemorrhage

**DOI:** 10.5811/cpcem.1574

**Published:** 2025-02-15

**Authors:** Jan Aldrin Enabore, Robert Vezzetti, Guyon Hill

**Affiliations:** * Dell Children’s Medical Center, Department of Emergency Medicine, Austin, Texas; † University of Texas Dell Medical School, Austin, Texas

**Keywords:** torticollis, pediatric, cerebellar hemorrhage, cavernoma, case report

## Abstract

**Introduction:**

There exists a wide differential of etiologies for pediatric torticollis that extends beyond musculoskeletal factors.

**Case Report:**

We present a novel case of a pediatric patient with an acute atraumatic hemorrhage of the left cerebellum presenting with gradual worsening torticollis. Upon further diagnostic workup, he was found to have an intracerebral hemorrhage due to a cerebellar cavernous malformation. Although the hemorrhage boundaries were extensive, the patient had only exhibited transient dysmetria and facial weakness, with ultimate resolution of torticollis and these neurological symptoms after several days.

**Conclusion:**

This case demonstrates the importance of maintaining a broad differential in the workup of acute pediatric torticollis.

## INTRODUCTION

The etiology of acute pediatric torticollis is most often either traumatic, infectious, or medication induced.^1^ However, keeping a broad differential in evaluating these children is always important, as the etiologies can be vast and include potentially life-threatening causes. A lack of diagnosis and failure of therapeutic interventions should always spur further workup. We present a novel case of a pediatric patient with an acute atraumatic cerebellar hemorrhage secondary to cavernous malformation presenting with isolated torticollis.

## CASE REPORT

An eight-year-old male without chronic medical problems presented to the emergency department (ED) after waking up with posterior left neck pain and an inability to move his neck to the left side. The mother believed he had slept in an uncomfortable position the previous evening and denied any history of recent trauma. The patient had no history of fevers and no prior similar symptomatology. In the preceding week, the patient had episodes of nausea, vomiting, and headache, but all these symptoms had since resolved several days prior. At the time of presentation, there were no complaints of significant headache, nausea, gastrointestinal symptoms, vision changes, focal weakness, or difficulty ambulating. No recent medications were started.

The initial vitals were as follows: temperature 37.6° Celsius, blood pressure 102/72 millimeters of mercury (mm Hg), heart rate 98 beats per minute, respiratory rate 24 breaths per minute, and oxygen saturation 98% on room air. On physical examination, the child was calm and in no significant distress. He held his head and chin deviated to the right and in slight cervical extension. He had left lateral neck tenderness over the sternocleidomastoid region but no posterior midline tenderness. There were no identified concerns for infection, mass, or lymphadenopathy. He had baseline right eye strabismus, but extraocular muscles were intact bilaterally without visual field deficits. An oropharyngeal exam revealed mild bilateral tonsillar swelling; however, there was no evidence of uvula deviation, peritonsillar abscess, sublingual tenderness, submandibular swelling, or dental infection. He could ambulate and follow all commands but did so with an unchanged head and neck position. He also had equal strength and sensation in all extremities. There were no other physical exam abnormalities identified.

**Image 1:**
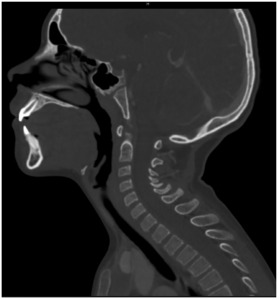
Sagittal view of computed tomography soft tissue neck with contrast with evidence of maxillary sinus disease and without evidence of hemorrhage or other infectious etiology for torticollis.

Laboratory findings for complete blood count, chemistry, C-reactive protein, and creatine kinase were within normal limits. However, his erythrocyte sedimentation rate was elevated: 38 millimeters per hour (mm/hr) (reference range: 0–20 mm/hr). A computed tomography (CT) with intravenous (IV) contrast of his neck showed only mild maxillary sinus disease and no other documented abnormalities (Image 1). Based on these findings, a trial of supportive treatment was undertaken consisting of oral acetaminophen, IV ketorolac, and two doses of IV diazepam.

**Image 2:**
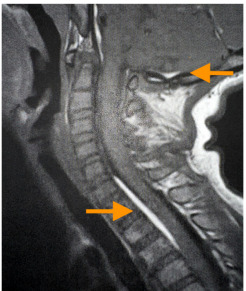
Sagittal view of magnetic resonance imaging of the neck with contrast demonstrating left cerebellar hemorrhage (upper arrow) with fifth cervical to second thoracic spinal extension (lower arrow).

**Image 3:**
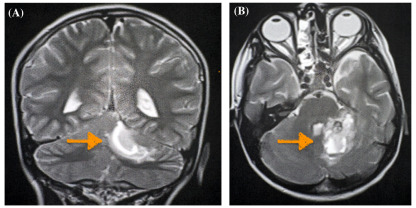
Magnetic resonance imaging of the brain with contrast, (A) coronal view and (B) axial view, demonstrating a left cerebellar hemorrhage secondary to a cavernous malformation (arrows).

Given that there was no significant improvement, magnetic resonance imaging (MRI) of the cervical spine was obtained and revealed an epidural spinal hemorrhage and a partially visualized left cerebellar hemorrhage. Subsequent imaging included an MRI brain with contrast and a CT angiogram that showed a left cerebellar hemorrhage from a cavernous malformation without evidence of an intracranial mass (Images 2, 3). The patient remained in the intensive care unit for five days, where he transiently developed left facial weakness and left upper extremity dysmetria. He received a course of dexamethasone and pain medications during this time. Pediatric neurosurgery discussed surgical resection options; however, the family deferred in favor of close neurosurgical follow-up. His torticollis and neurological deficits were resolved by the time of discharge on day five of hospitalization.

*Population Health Research Capsule*

What do we already know about this clinical entity?

*Acute pediatric torticollis is attributed to trauma, infection, or medication. Many cerebrovascular malformations are incidentally discovered and remain asymptomatic*.

What makes this presentation of disease reportable?

*This rare presentation of isolated torticollis resulted from an atraumatic cerebellar hemorrhage secondary to cerebrovascular malformation*.

What is the major learning point?

*It is important to comprehensively investigate unresolved pediatric torticollis*.

How might this improve emergency medicine practice?

*Emergency physicians should maintain a broad differential and consider advanced imaging, such as magnetic resonance imaging, when initial evaluations are inconclusive*.

## DISCUSSION

Torticollis arises from the Latin roots of “torus” and “collum,” meaning twisted and neck, respectively. It is sometimes also referred to as cervical dystonia. This is a common disorder that can occur at all ages. The most common cause of torticollis is congenital muscular torticollis, typically caused by a hematoma or fibroma on the sternocleidomastoid muscle that regresses by eight months of age.^2^ However, the causes of acquired torticollis are vast. Traumatic, infectious, and medication-induced torticollis are the three most common causes of acute torticollis. Common infectious etiologies include retropharyngeal and peritonsillar abscesses and cervical lymphadenitis. Common medication culprits include cholinergic and antipsychotic medications.^1^

A thorough clinical history and physical examination can frequently lead to a concise workup for acute cervical torticollis in the pediatric patient. In traumatic cases without neurological findings, plain-film anterior-posterior and lateral cervical spine radiographs are appropriate and can be followed by CT when non-diagnostic.^3^ When superficial masses are present in the cervical region, ultrasound can be used to limit exposure to ionizing radiation. When clinical signs and symptoms of infection are present, a CT with IV contrast is the appropriate test.^1^,^4^ This can determine the extent and location of the infection, such as in cases of retropharyngeal abscess, peritonsillar abscess, lymphadenitis, or similar skin and soft tissue infections in the paracervical and perioral regions.

This patient’s presentation did not yield a diagnosis in the ED, nor did he respond as expected to analgesia and muscle relaxants. The patient was admitted to obtain an MRI, which showed an acute cerebellar hemorrhage with ipsilateral cervical extension. This led to the discovery of a cerebral cavernous malformation (CCM) in the cerebellum. Cavernous malformations are a conglomeration of abnormal, intermixed small and large blood vessels and are also referred to as cavernomas, cavernous angiomas, cavernous hemangiomas, or intracranial vascular malformations. The etiology of these malformations is unknown, but it is suspected that there is likely a genetic component.^5^ Magnetic resonance imaging has nearly 100% sensitivity for detecting a CCM, and many are incidentally discovered.^6^ The decision for surgical resection of a CCM depends on size, location, and symptomatology.^5^ Ultimately, this patient was treated with steroids and analgesics with an improvement of his torticollis and was discharged five days after the initial diagnosis.

Briefly, this case serves as a great example of pursuing continued workup of acute, unresolved torticollis, especially when the most common culprits of trauma, infection, and medication-induced causes have been ruled out. There are two similar case reports of isolated torticollis in the pediatric population: a nine-year-old after falling off her bicycle with brain stem cavernoma hemorrhage and an eight-month-old with a large cerebellar-pontine angle arachnoid cyst.^7^,^8^ However, to our knowledge, this is the first documented case of isolated torticollis secondary to atraumatic cerebellar hemorrhage from a cavernous malformation.

## CONCLUSION

This pediatric case report highlights the importance of a comprehensive approach in assessing acute torticollis in children. While factors like trauma, infection, and medication-related issues are frequently encountered, the presented case unveils an atraumatic cerebellar hemorrhage secondary to a cavernous malformation as the root cause. Despite the extensive hemorrhagic involvement, the patient’s symptoms were initially confined to torticollis, later accompanied by transient neurological manifestations that ultimately resolved through conservative management. This case emphasizes the ongoing need for a thorough investigation of unresolved torticollis, emphasizing the potential for life-threatening causes within the diverse spectrum of pediatric presentations.
